# A Monte Carlo Analysis of Actual Maximum Exposure From a 5G Millimeter-Wave Base Station Antenna for EMF Compliance Assessments

**DOI:** 10.3389/fpubh.2021.777759

**Published:** 2022-01-07

**Authors:** Bo Xu, David Anguiano Sanjurjo, Davide Colombi, Christer Törnevik

**Affiliations:** Ericsson Research, Ericsson AB, Stockholm, Sweden

**Keywords:** 5G, base station antenna, beamforming, EMF exposure, incident power density, millimeter wave

## Abstract

International radio frequency (RF) electromagnetic field (EMF) exposure assessment standards and regulatory bodies have developed methods and specified requirements to assess the actual maximum RF EMF exposure from radio base stations enabling massive multiple-input multiple-output (MIMO) and beamforming. Such techniques are based on the applications of power reduction factors (PRFs), which lead to more realistic, albeit conservative, exposure assessments. In this study, the actual maximum EMF exposure and the corresponding PRFs are computed for a millimeter-wave radio base station array antenna. The computed incident power densities based on near-field and far-field approaches are derived using a Monte Carlo analysis. The results show that the actual maximum exposure is well below the theoretical maximum, and the PRFs similar to those applicable for massive MIMO radio base stations operating below 6 GHz are also applicable for millimeter-wave frequencies. Despite the very low power levels that currently characterize millimeter-wave radio base stations, using the far-field approach can also guarantee the conservativeness of the PRFs used to assess the actual maximum exposure close to the antenna.

## Introduction

To meet the increasing demands on mobile traffic data, the fifth-generation cellular communication technology (5G) exploits the frequency spectrum above 24 GHz, which provides much wider and contiguous bands compared with the crowded and fragmented spectrum below 6 GHz. This frequency spectrum is also called millimeter-waves (mmW) or the frequency range 2 (FR2) in 5G (1). Before radio base stations (RBSs) are placed on the market, manufacturers normally need to conduct electromagnetic field (EMF) exposure assessments to determine the so-called compliance boundaries or exclusion zones. Outside the compliance boundary, the EMF exposure from an RBS is below the relevant EMF exposure limits, for example, those provided in the international EMF exposure guidelines. The most widely adopted EMF exposure guidelines are provided by the International Commission on Non-Ionizing Radiation Protection (ICNIRP) (2, 3). As the previous generations of mobile communication technologies, 5G equipment, including mmW RBSs, must comply with the same EMF exposure guidelines.

EMF exposure assessments for RBSs are normally carried out using the far-field antenna radiation patterns (4). This approach, referred to as the far-field approach in the rest of the article, is accurate when the resulting compliance boundary is located sufficiently far from the RBS antenna. When the transmitted power of an RBS is very low, the EMF exposure levels might be greatly overestimated by the far-field approach (5). In such scenarios, estimation formulas based on cylindrical models (6), full-wave simulation methods, and field strength measurements (7) are usually used (4) to get more accurate compliance boundaries.

For RBSs enabling multi-input multi-output (MIMO), massive MIMO, and beamforming, the antenna radiation pattern changes dynamically according to the real-time channel conditions. When determining the compliance boundary using maximum configured power and the envelope of all possible radiation patterns, the results are very conservative. This is because such an approach assumes that all the power is constantly transmitted in all directions without considering the effects of time-averaging. The EMF exposure determined based on these unrealistic assumptions is referred to as the theoretical maximum exposure. Therefore, the International Electrotechnical Commission (IEC) (4) has developed methodologies that allow for more accurate exposure assessments based on the actual maximum exposure. This approach is described in an IEC technical report (8), and it will be part of the new edition of the international standard IEC 62232 (9), which is currently under revision. The actual maximum exposure considers the effects of dynamical radiation pattern changes on the time-averaged EMF levels and can be determined by applying a power reduction factor (PRF) to the theoretical maximum transmitted power. In literature, the PRF is normally determined by statistically conservative models, for instance, based on the 95th percentile time-averaged exposure derived from the cumulative distribution function (CDF) (10–14). Results from recent measurement campaigns (15–17) also show that the EMF exposure from real massive MIMO RBS sites is well below the actual maximum exposure derived from the statistical models.

Unlike massive MIMO RBSs operating below 6 GHz, which are characterized by high-peak equivalent isotropically radiated power (EIRP) and support wide coverage in ranges of a few kilometers, mmW RBSs usually aim at providing smaller cell coverage in a radius of a few hundred meters but with higher capacity. Therefore, the peak EIRP levels of mmW RBSs are usually lower, resulting in a smaller compliance boundary with a typical front compliance distance of a few meters or less. When applied very close to the antenna, the estimation of exposure levels using the far-field approach may be very conservative. In addition, it is not clear whether the PRFs derived from the far-field radiation patterns obtained in some other studies (10–14) are still applicable when compliance boundaries are in the radiating near-field region. Several works, for example, Refs (18–31), addressing the EMF compliance and assessments for lower-power mmW devices can be found in literature, but only a few (32) address the actual maximum exposure from mmW RBSs.

This article presents a case study of the actual maximum exposure for a typical mmW RBS antenna configuration. The Monte Carlo method is applied to the time-averaged exposure using a predefined user equipment (UE) distribution and a beamforming codebook. The actual maximum exposure and the PRF in the radiating near-field region are derived through full-wave simulations. The results are also compared with the actual maximum exposure computed using the far-field approach.

## Methods

### Array Antenna Model and Beam Patterns

**Figure 1:**
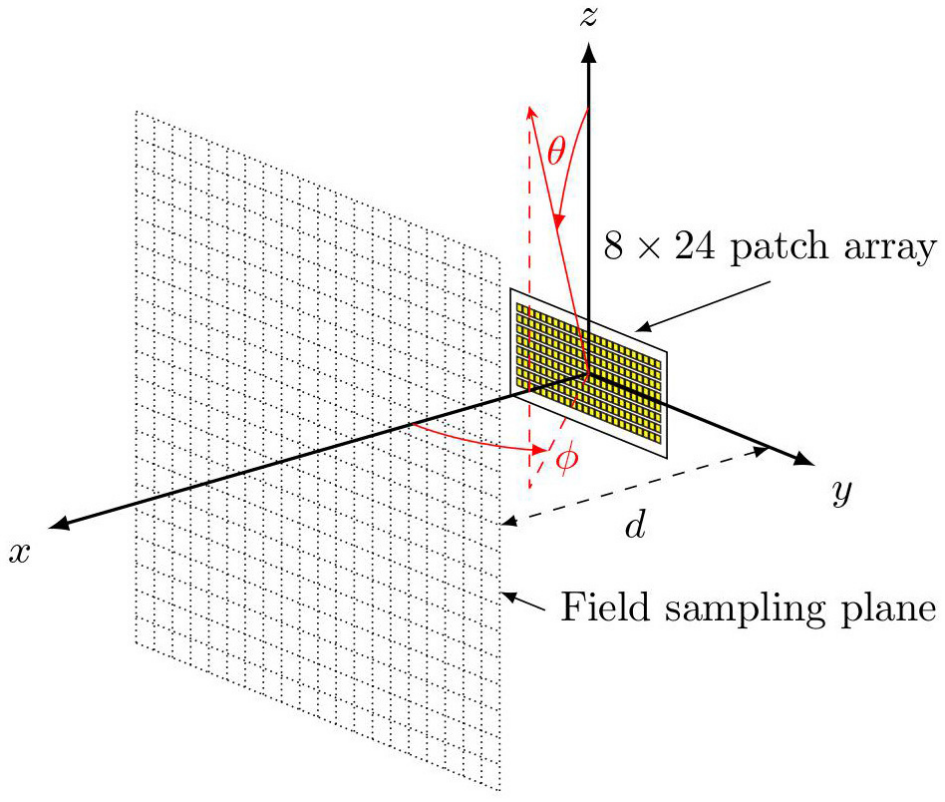
Feko model of the 8 × 24 patch antenna array. The red markers indicate the convention of the used spherical coordinate system.

**Figure 2:**
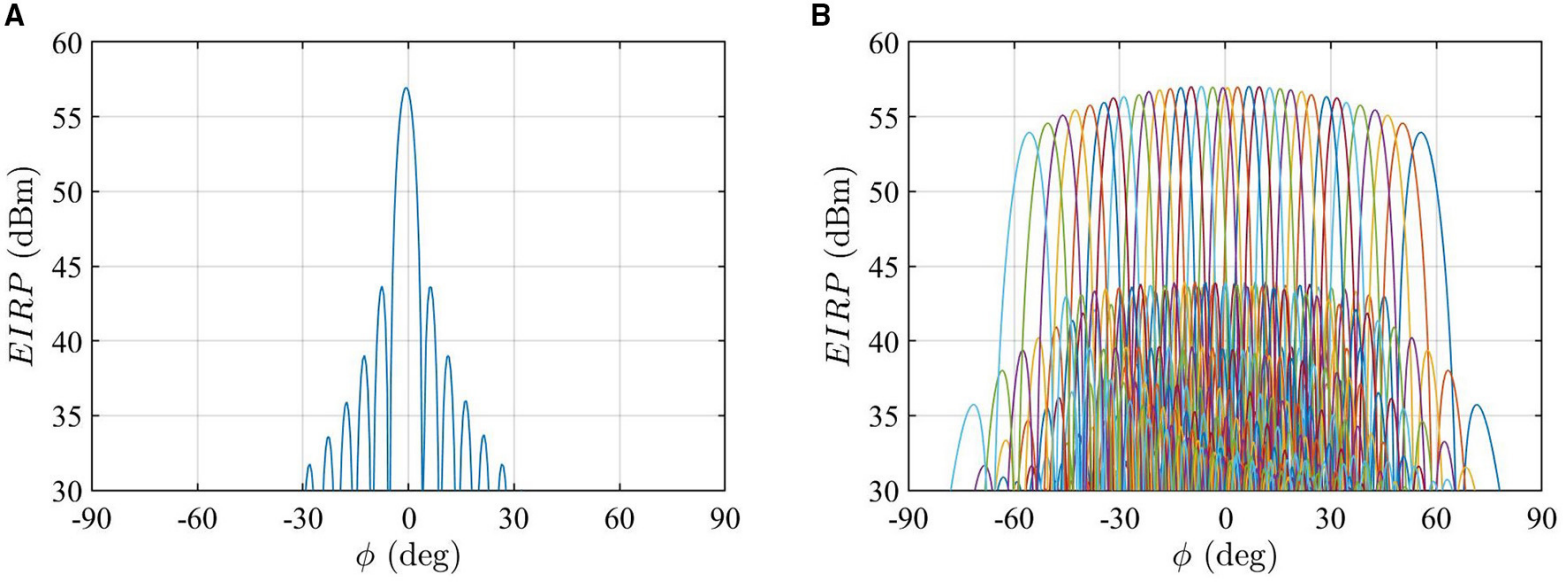
**(A)** The azimuthal cut of the radiation pattern for the beam pointing at $\theta_{0}=93^{{}^{\circ}}$ and ϕ = −1°, which is the closest beam to the broadside direction in the used codebook. **(B)** The azimuthal cut of simulated beam patterns at $\theta_{0}=93^{{}^{\circ}}$, which are those used in the Monte Carlo analysis. The beam patterns with different colors from the left to the right correspond to beam indices *l* denoted from 1 to 34.

In this study, an 8 × 24 patch array is considered as the mmW RBS antenna using the model built in Ref (33), as shown in Figure 1. The array antenna operates at 28 GHz. A predefined codebook based on progressive phase shift is created to enable the spatial coverage of 30 degrees in elevation and 120 degrees in azimuth. As the UE distribution considered below is set at θ = 93° and spread over the azimuthal plane [see Equation (5)], only the beams pointing at the plane of θ = 93° contribute to the Monte Carlo analysis. The used beam patterns, in EIRP, are shown in Figure 2. As an example, the azimuthal cut of the far-field pattern for the beam closest to the broadside direction is shown in Figure 2A, and the far-field patterns for the beams pointing at the cut of θ = 93° are shown in Figure 2B. For simplicity, only one polarization is used, and the fields generated by the orthogonal polarization is conservatively summed up by adding 3 dB to the peak EIRP. The combined peak EIRP level is 58.4 dBm. A time division duplex (TDD) downlink duty cycle of 75% is used. The peak total EIRP is thus (58.4 + 10log_10_0.75) dBm. The field strength distributions on different planes and the radiation pattern for each beam are computed using the full-wave simulation software Altair Feko with the multilevel fast multipole method (MLFMM) solver. The electric field and magnetic field are sampled with a 5 mm interval on a 2 m × 2 m area in the *yz*-planes at different distances. The far-field radiation pattern for each beam is sampled with 0.5 degrees over the sphere.

### EMF Exposure Metrics

The EMF exposure metric used in this study is incident power density. For short, it is referred to as power density in the following. The power density limits are intended as spatial average and time average values according to the ICNIRP 1998 guidelines (2) and the ICNIRP 2020 guidelines (3). The implications on EMF assessments of RBSs between the ICNIRP 1998 and ICNIRP 2020 guidelines are addressed in a separate article (34). Hereafter, only the differences relevant to the investigated mmW RBS are presented.

For the ICNIRP 1998 guidelines, the power density is averaged over any 20 cm^2^ of exposed area and over 68*f*^−1.05^ min where *f* is the frequency in GHz (about 2 min and 3 s at 28 GHz.), while for the ICNIRP 2020 guidelines, the power density at 28 GHz is averaged over a 4 cm^2^ square and over 6 min. For the ICNIRP 1998 limits, the power density is averaged over square-shaped areas in this article. The applicable general public limit values are 10 W/m^2^ and 55*f*^−0.177^ W/m^2^ (about 30.5 W/m^2^ at 28 GHz) for the ICNIRP 1998 and ICNIRP 2020 guidelines, respectively.

When assessing compliance with the ICNIRP 2020 guidelines and for the power levels used in this article (lower than 1 W), the local exposure is the limiting factor determining the compliance boundary. Compliance with the whole-body exposure limits is implicitly met using the whole-body exclusion criteria^1^. The mentioned power density limits and requirements on spatial averaging and time averaging are summarized in Table 1.

**Table 1:** Incident power density limits for the general public at 28 GHz and assumed number of active UEs during the averaging time.

	**Incident power density limits for local exposure**	**Averaging area**	**Averaging time**	**Assumed number of active UEs during EMF averaging time for Monte Carlo analysis**
ICNIRP 1998	10 W/m^2^	20 cm^2^	2 min and 3 s	*N* = 33, 66, 100
ICNIRP 2020	30.5 W/m^2^	4 cm^2^	6 min	*N* = 100, 200, 300

The spatially averaged power density is expressed as


(1) $$S=\frac{1}{A}\int_{A}\text{Re}\left[E\times H^{*}\right]\cdot \hat{n}\text{ d}A,$$

where *A* is the averaging area in m^2^; ***E*** and ***H*** are the root-mean-square (rms) electric and magnetic fields in V/m and A/m, respectively; ^*^ denotes the conjugate; and $\hat{n}$ is the unit vector normal to *A*. This expression is in line with the definition in Refs (35, 36). In this article, the spatially averaged power density is computed in planes parallel to the antenna surface and for different distance *d* (see Figure 1). This is referred to as the near-field approach in the following.

When applying this expression on devices operating close to the human body, such as 5G mobile phones using mmW bands, the assessment plane and the average areas parallel to the outer surface of the device are mostly relevant. For distances up to a few meters of interest in this study, the orientation of the averaging area may be arbitrary considering the real usage scenarios. Thus, for mmW RBSs, the magnitude of the Poynting vector may also be a reasonable quantity for EMF assessments


(2) $$\begin{array}{l} S=\frac{1}{A}\underset{A}{\int }|\text{Re}\left[E\times H^{*}\right]|\text{ d}A. \end{array}$$

The results using Equation (1) are presented in the main body of the article, whereas the comparison between Equations (1) and (2) for actual maximum power density is given in the Supplementary Material. The differences in actual maximum power density are found to be small (<5%, see Supplementary Figure 1).

In the far-field region, the power density is well characterized by the spherical model (also called the far-field formula) (4). In a spherical coordinate system, the power density can be expressed as


(3) $$\begin{array}{l} S\left(r,\theta ,\phi \right)=\frac{PG\left(\theta ,\phi \right)}{4\pi r^{2}}=\frac{EIRP\left(\theta ,\phi \right)}{4\pi r^{2}}, \end{array}$$

where *P* is the transmitted power in watts, *G* is the radiation gain in linear scale, *r* is the radius in meters, θ is the polar angle in degrees, and ϕ is the azimuthal angle in degrees. In the far-field region, the fields are uniform over the averaging area, and Equation (3) can also be considered to provide the spatially averaged results.

In the ICNIRP 2020 guidelines, in addition to the time-averaged power density, the so-called brief exposure limits are defined for local exposure and apply to any pulse, group of pulses, or subgroup of pulses in a train, as well as from the summation of exposures (including non-pulsed EMFs), occur within 6 min. The brief exposure limits corresponding to incident power density limits are given in terms of incident energy density. Between >6 and 300 GHz, the incident energy density limits in kJ/m^2^ for the general public are expressed as


(4) $$\begin{array}{l} U\left(t\right)=55f^{-0.177}\times 0.36\left[0.05+0.95{\left(t/360\right)}^{0.5}\right] \end{array}$$

where *f* is the frequency in GHz and *t* is time in seconds. *U* is to be averaged over 4 cm^2^ square at 28 GHz as for power density. When *t* = 360 *s*, Equation (4) gives the same value as if integrating the power density limits (see Table 1 for the ICNIRP 2020 guidelines) over 6 min.

In this study, the actual maximum exposure levels and PRFs are derived using Equations (1) and (3) with the following conditions.

### Service Probability

**Figure 3:**
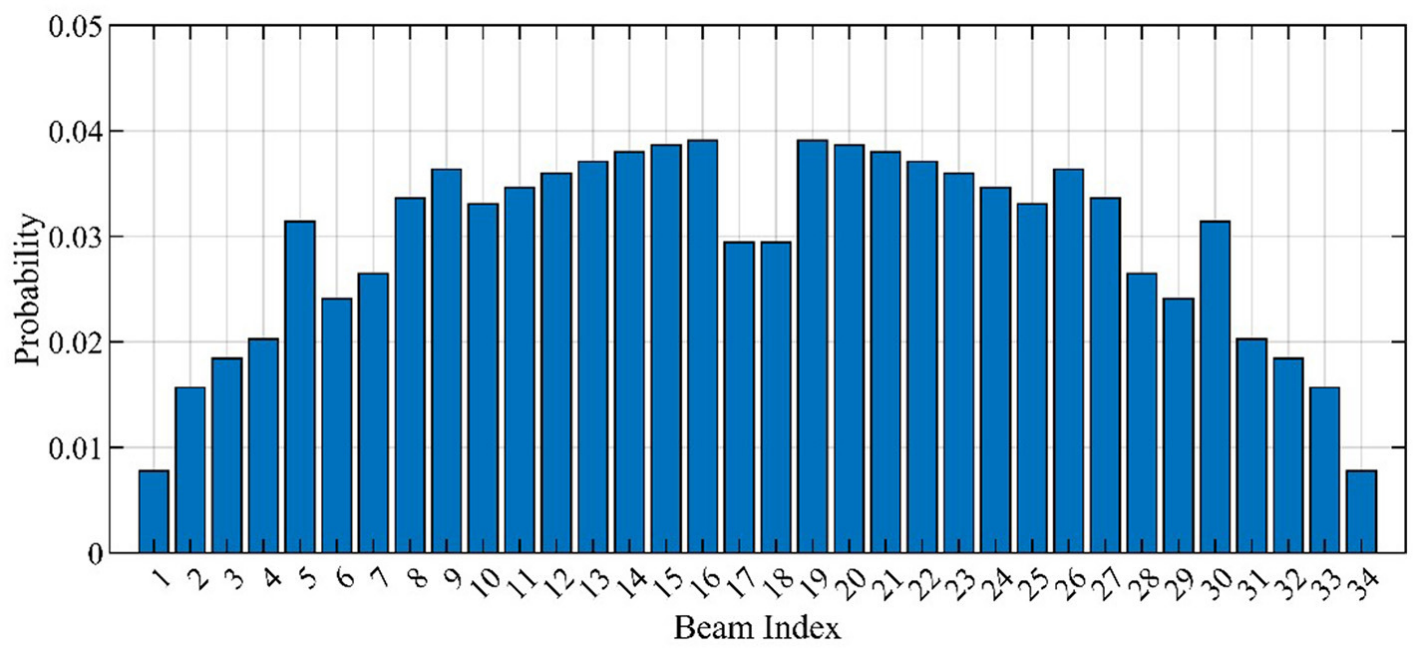
Service probabilities of the selected beams.

**Figure 4:**
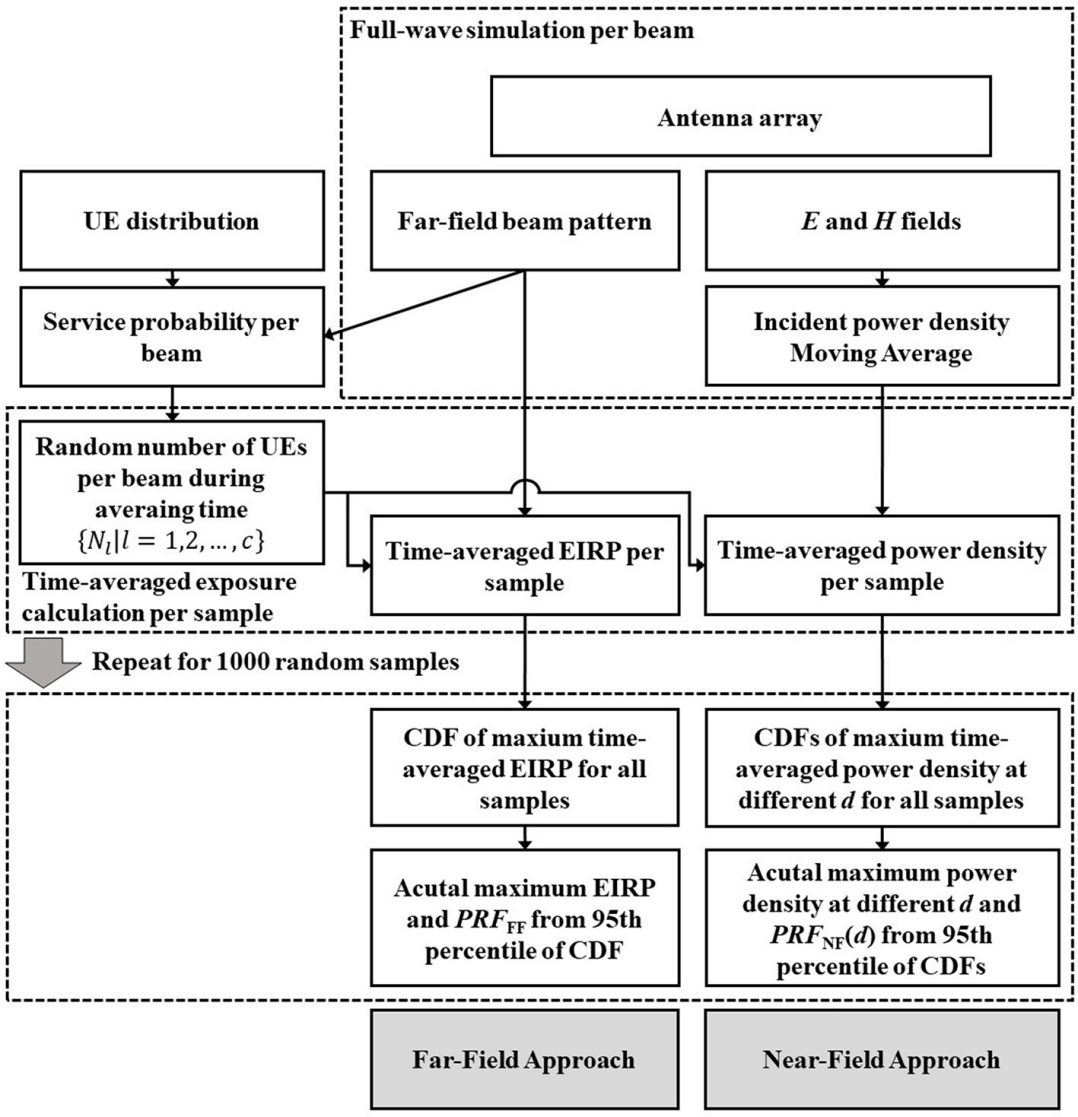
Workflow of antenna simulation and Monte Carlo analysis.

For a statistical approach, the service probability of each beam depends on the distribution of active UE in space. In this study, the spatial UE distribution defined in Refs (10) and (14) is reused, which is a cosine-shaped function:


(5) $$w\left(\theta ,\phi \right)=\{\begin{array}{cc} \frac{3}{4}\delta \left(\theta -\theta_{0}\right)\cos\frac{3\phi }{2}, & -60^{{}^{\circ}}\leq \phi \leq 60^{{}^{\circ}}\\ 0, & \text{otherwise} \end{array}$$

where $\theta_{0}=93^{{}^{\circ}}$ for the used beams. Such a distribution can be found in Figure 3 of (10) and Figure 4 of (14). It implies that more UEs are located close to the boresight direction ($\underset{\theta }{\int }w\left(\theta ,0^{{}^{\circ}}\right)\text{d}\theta =\frac{3}{4}$) and fewer UEs are located close to the angular edge of the cell ($\underset{\theta }{\int }w\left(\theta ,\pm 60^{{}^{\circ}}\right)\text{d}\theta =0$). This distribution conservatively assumes that the UEs are only distributed in the azimuthal direction, and no beam scanning in elevation is therefore applied during the Monte Carlo analysis. Consequently, the derived PRFs and actual maximum exposure are more conservative than those derived with beam-steering in elevation, as pointed in Ref (10).

Similar to those in Refs (10) and (14), the angular service establishment range for each beam is defined as


(6) $$\begin{array}{l} \left({\hat{\theta }}_{l},{\hat{\phi }}_{l}\right)=\left\{\left(\theta ,\phi \right)|EIRP\left(\theta ,\phi ,l\right)>EIRP\left(\theta ,\phi ,m\right),l\neq m\right\},\\ l,m=1,2,\cdots \;,c, \end{array}$$

where *l* and *m* are the beam indices, and the total number of beams is denoted by *c*.

The service probability of the *l*th beam is thus defined as (10, 14).


(7) $$p\left(l\right)=\int_{{\hat{\theta }}_{l}}\int_{{\hat{\phi }}_{l}}w\left(\theta ,\phi \right)\sin\theta \text{ d}\theta \text{d}\phi .$$

Using the far-field beam patterns shown in Figure 2B and the UE distribution [Equation (5)], the service probabilities for the selected beams are shown in Figure 3. In Figure 3, the beam index from 1 to 34 corresponds to the beam pointing from −60 degrees to + 60 degrees. Note that the *p*(*l*) values for the beam indices 17 and 18 closest to the broadside direction (see Figure 3) are slightly lower than the adjacent beams. This is because the beams are denser in the broadside direction for the used codebook (see Figure 2) and the beams closest to the broadside direction usually have narrower beamwidth.

### Monte Carlo Analysis

The time-averaged EIRP and power density can be computed through the Monte Carlo method using Equation (7). Assuming that *N* active UEs are scheduled during the EMF averaging time, the number of UEs served by the *l*th beam, *N*_*l*_, during the EMF averaging time is determined by the multinomial distribution,


(8) $$\begin{array}{l} \mathrm{Pr}\left\{N_{l}|N\right\}\sim \text{Multinom}\left(N,\left\{p\left(l\right)\right\}\right),\;l=1,2,\ldots ,c \end{array}$$

where $N=\overset{c}{\underset{l=1}{\sum }}N_{l}$. This is aligned with what is used in Ref (14). The energy transmitted to each UE is assumed equal, and the traffic load is assumed to be 100% (full buffer). It is well understood that a smaller *N* will result in larger PRF and actual maximum exposure. In this study, *N* = 33, 66, 100 and *N* = 100, 200, 300 are assumed for the ICNIRP 1998 (averaged over 2 min and 3 s) and ICNIRP 2020 (averaged over 6 min) limits, respectively. According to the network counter data from real operating networks (15–17), these chosen *N* values are very conservative. See Table 1 for the summary of used limits and assumptions. The detailed description of Monte Carla analysis to address the implications of the incident energy density limits can be found in the Supplementary Material. For each sample used for the Monte Carlo analysis, the time-averaged EIRP can be written as


(9) $$\begin{array}{l} EIRP_{\text{av}}\left(\theta ,\phi \right)=\overset{c}{\underset{l=1}{\sum }}\frac{N_{l}}{N}EIRP_{l}\left(\theta ,\phi \right), \end{array}$$

where *EIRP*_*l*_(θ, ϕ) is the EIRP of the *l*th beam in direction (θ, ϕ).

For each sample, the peak time-averaged, spatially averaged power density in different planes *x* = *d* can be written as


(10) $$S_{\text{av}}\left(d\right)=\underset{y,z}{\text{max}}\sum_{l=1}^{c}\frac{N_{l}}{N}S_{l}\left(d,y,z\right),$$

where *S*_*l*_(*d, y, z*) is the spatially averaged power density on the plane of *x* = *d* for the *l*th beam.

For a given *N*, the Monte Carlo analysis is carried out using *EIRP*_av_(θ, ϕ) and *S*_av_(*d*) from 1000 samples. Different samples are generated with different combinations of {*N*_*l*_|*l* = 1, 2, …, *c*}. For a given *N*, The CDF of *EIRP*_av_ is calculated using the results of Equation (9) for all samples. The CDFs for the near-field approach are computed for different *d* and *N* with Equation (10). The workflow of the Monte Carlo analysis can be found in Figure 4. The PRF for the far-field approach is calculated using the 95th percentile of the CDF of *EIRP*_av_


(11) $$\begin{array}{l} PRF_{\text{FF}}\left(\theta ,\phi \right)=\frac{EIRP_{\text{av},0.95}\left(\theta ,\phi \right)}{EIRP_{\text{evlp}}\left(\theta ,\phi \right)} \end{array}$$

where *EIRP*_av,0.95_(θ, ϕ) is the 95th percentile in the CDF of *EIRP*_av_(θ, ϕ) in each direction and *EIRP*_evlp_(θ, ϕ) = max_*l*=1,2,…,*c*_*EIRP*_*l*_(θ, ϕ) is the envelope of traffic beam patterns in terms of EIRP. In the following, the PRF for the far-field approach is referred to as the maximum *PRF*_FF_(θ, ϕ) value over the scan range, *PRF*_FF,m_, which is chosen to determine the compliance distance in the following.

In the near-field approach, the PRF at each distance *d* is calculated using the 95th percentile of the CDF of *S*_av_(*d* ).


(12) $$PRF_{\text{NF}}(d)=\frac{S_{\text{av},\text{0}.\text{95}}\left(d\right)}{\underset{l,y,z}{\text{max}}S_{l}\left(d,y,z\right)}$$

where *S*_av,0.95_(*d*) is the 95th percentile in the CDF of *S*_av_(*d* ).

### Calculation of Compliance Distance in Front of RBS Antenna

For communication purposes, simplified compliance boundaries, for example, the box-shaped compliance boundary (4), are usually used instead of iso-surface compliance boundaries. In this study, the compliance distance in front of the RBS is of interest. For the near-field approach, the compliance distance *CD*_NF,*x*_ for the actual maximum exposure in front of RBS antenna can be calculated by


(13) $$\begin{array}{l} CD_{\text{NF},\text{x}}=\min d,\text{ for }S_{\text{av},0.95}\left(d\right)\leq S_{\lim} \end{array}$$

where *S*_lim_ is the power density limit.

In general, the spherical model (or the far-field formula) determining the compliance boundary for actual maximum exposure can be written as


(14) $$\begin{array}{l} r\left(\theta ,\phi \right)=\sqrt{\frac{EIRP_{\text{evlp}}\left(\theta ,\phi \right)\times PRF_{\text{FF},\text{m}}}{4\pi S_{\lim}}}. \end{array}$$

The front distance of the box-shaped compliance boundary can be expressed as


(15) $$CD_{\text{FF},x}=\underset{\theta ,\phi }{\text{max}}r_{x}\left(\theta ,\phi \right),$$

where *r*_*x*_(θ, ϕ) is *r*(θ, ϕ) projected to the *x*-axis.

## Results

**Figure 5:**
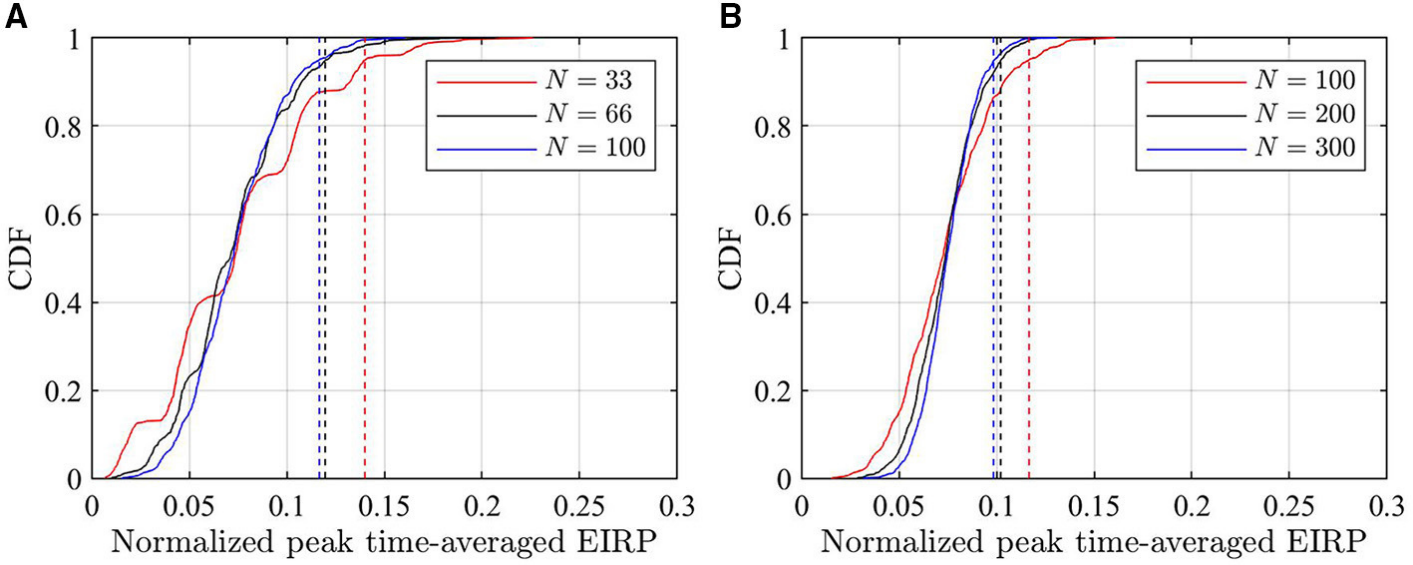
CDFs of peak time-averaged EIRP normalized to the maximum EIRP using different *N* samples. **(A)** CDF considered for ICNIRP 1998 limits. **(B)** CDF considered for ICNIRP 2020 limits. The dashed lines indicate the corresponding 95th percentile.

**Figure 6:**
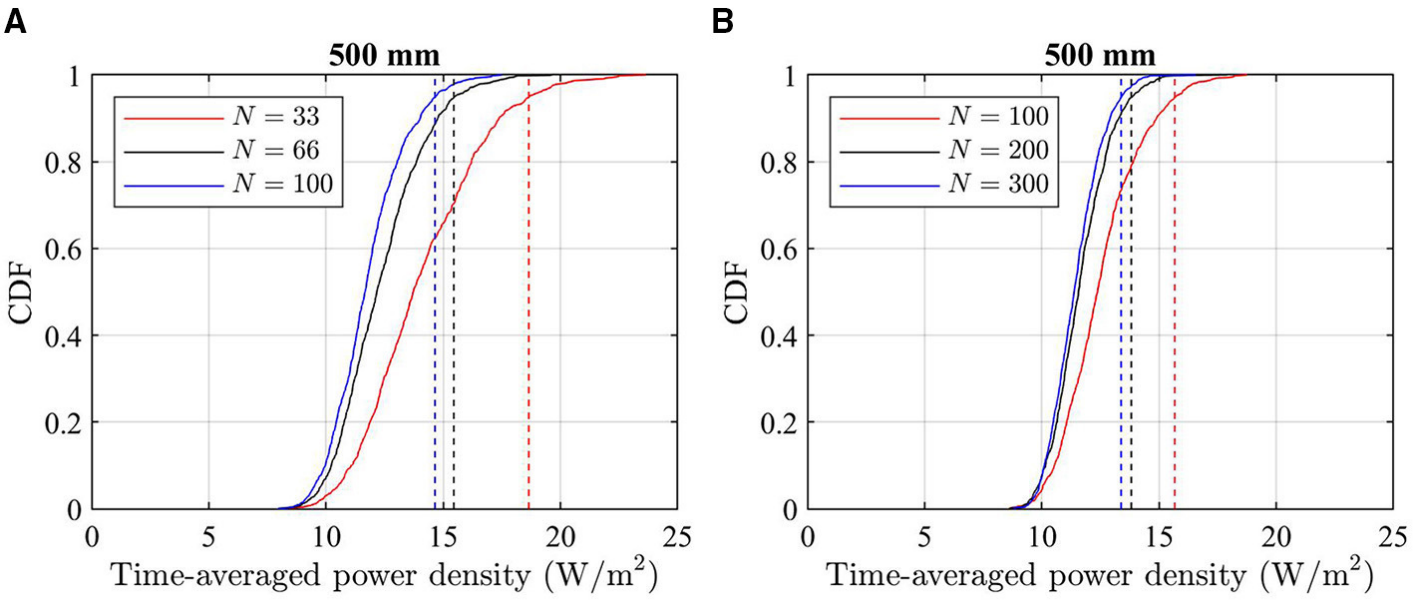
Examples of CDFs of peak time-averaged, spatially averaged power density for different *N* at *d* = 500 mm. **(A)** Peak 20 cm^2^ averaged power density considered for ICNIRP 1998 guidelines. **(B)** Peak 4 cm^2^ averaged power density considered for ICNIRP 2020 guidelines. The dashed lines indicate the corresponding 95th percentile.

The CDFs of *EIRP*_av_ [Equation (9)] in the maximum EIRP direction and an example of CDFs for peak time-averaged incident power density [Equation (10)] with different *N* are shown in Figures 5, 6, respectively. In Figure 5, the peak time-averaged EIRP is normalized to the maximum peak EIRP value of all used beams. It is clear that a smaller *N* results in larger 95th percentile of time-averaged EIRP and incident power density. This is well aligned with the findings of other works. As a conservative approach, *N* = 33 and *N* = 100 are selected in the following for ICNIRP 1998 and ICNIRP 2020 guidelines, respectively.

**Figure 7:**
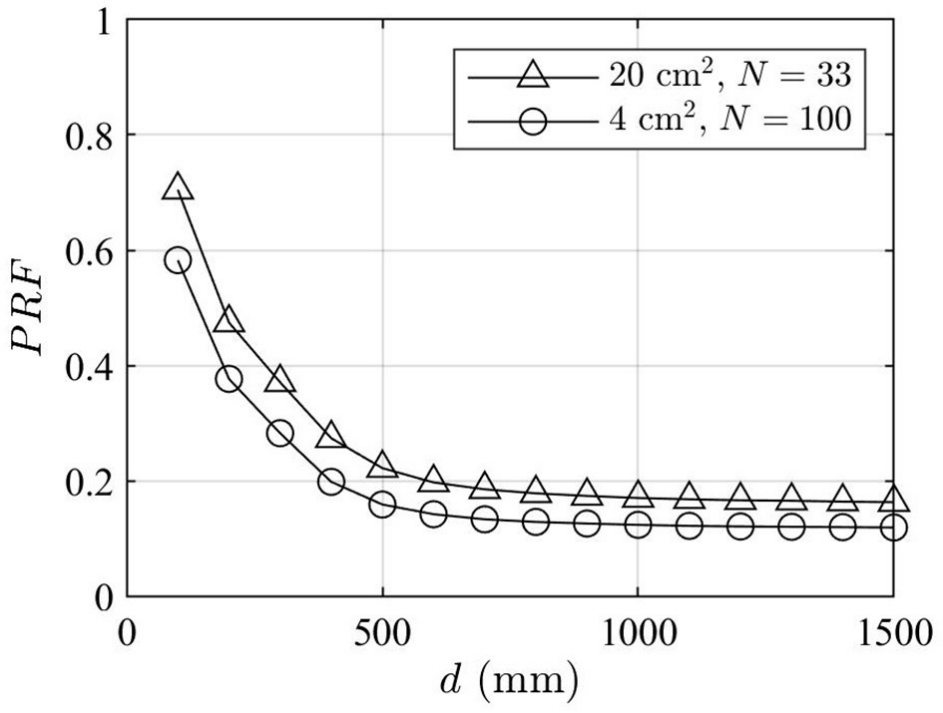
PRFs vs. distance obtained from the near-field approach.

Figure 7 shows the PRFs for 4 cm^2^ averaged and 20 cm^2^ averaged power density obtained from the near-field approach. As can be seen from the figure, the PRF values decrease with *d*. Good converges to the PRF obtained from the far-field approach can be observed above 0.5 m, and above 1 m, the difference in PRFs obtained from the near-field and the far-field approaches is negligible.

**Figure 8:**
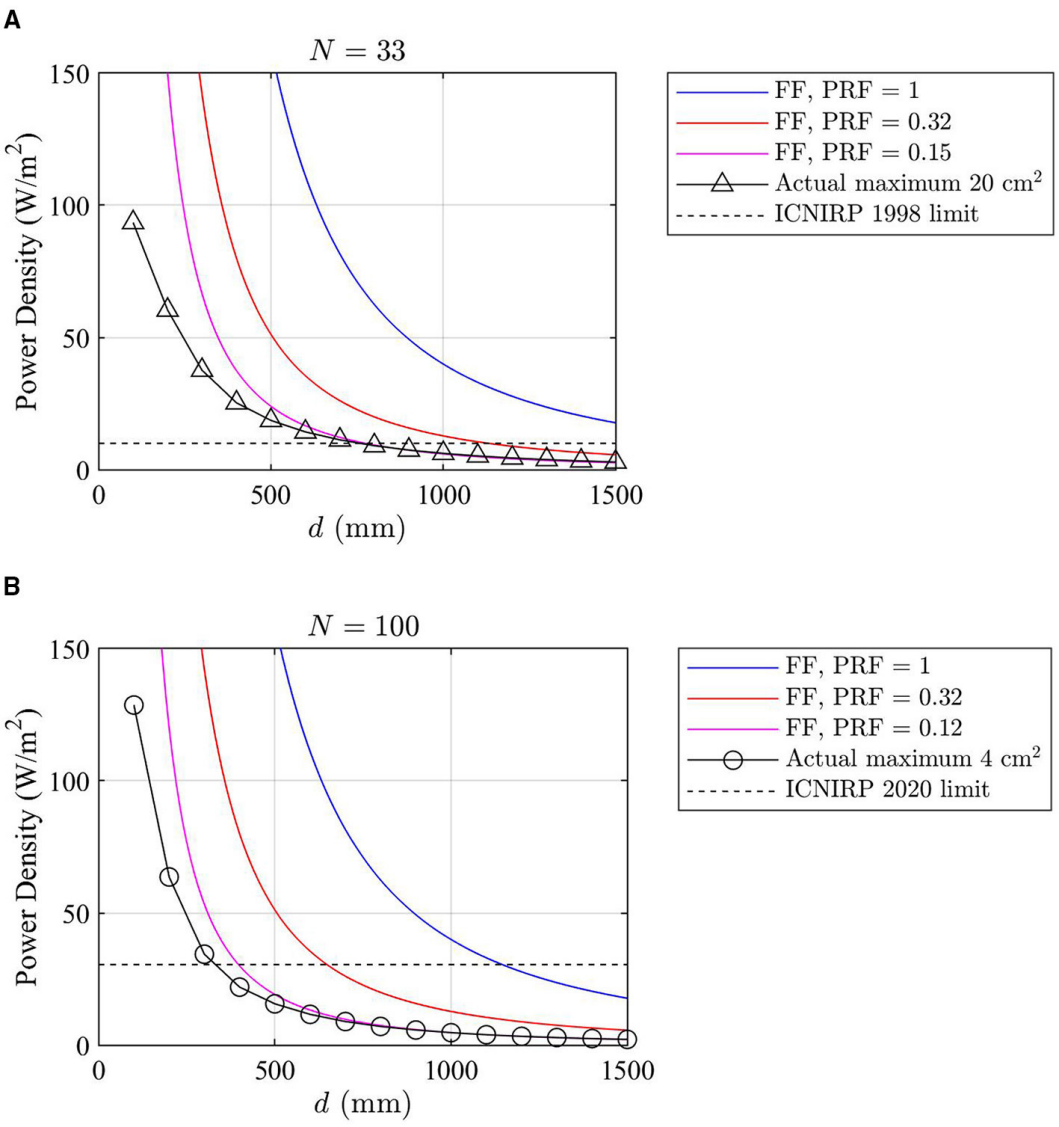
The blue curves indicate the power density levels obtained for the theoretical maximum exposure, i.e., PRF = 1, using the far-field approach. The orange curves indicate the power density levels by applying PRF = 0.32 using the far-field approach. In **(A)**, the pink curve indicates the power density levels by applying PRF = 0.15 derived from the far-field approach considered for the ICNIRP 1998 limits; the black solid line with the triangular markers is the actual maximum exposure obtained from the near-field approach averaged over 20 cm^2^; the black dashed line indicates the ICNIRP 1998 general public power density limit. PRF = 0.15 derived from the far-field approach and the actual maximum exposure derived from the near-field approach are based on *N* = 33 in the Monte Carlo analysis. In **(B)**, the pink curve indicates the power density levels by applying PRF = 0.12 derived from the far-field approach considered for the ICNIRP 2020 local exposure limits; the black solid line with the circular markers is the actual maximum exposure obtained from the near-field approach averaged over 4 cm^2^; the black dashed line indicates the ICNIRP 2020 local power density limit for the general public. PRF = 0.12 derived from the far-field approach and the actual maximum exposure derived from the near-field approach are based on *N* = 100 in the Monte Carlo analysis.

In Figure 8, the power density levels in front of the array obtained using different approaches are compared at distances below 1.5 m. The power density levels computed using the far-field formula [Equation (3)] are determined for different PRF values, including PRF = 1, that is, the theoretical maximum exposure condition, PRF = 0.32, that is, the PRF value recommended in Ref (10) for sub-6 GHz massive MIMO RBSs, and PRF = 0.15 (for ICNIRP 1998 limits) or PRF = 0.12 (for ICNIRP 2020 limits), that is, the PRF values obtained from the far-field approach shown in Figure 5. The actual maximum power densities averaged over 4 and 20 cm^2^, that is, *S*_av,0.95_(*d*), obtained from the near-field approach are also shown in Figure 8. The actual maximum power densities computed using the near-field approach are always smaller than or equal to those computed using the far-field approach.

**Figure 9:**
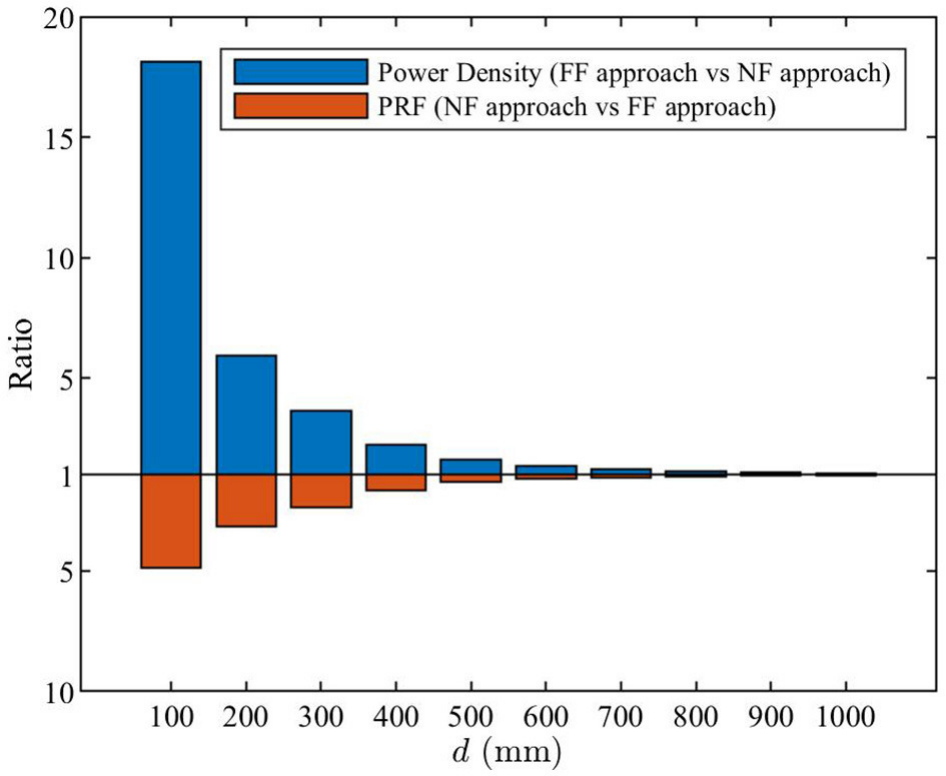
The blue bars are the ratios of theoretical maximum power density obtained using the far-field approach [Equation (3)] to the near-field approach [Equation (1)]. The red bars are the ratios of PRF obtained using the near-field approach [*PRF*_NF_(*d*) in Equation (12)] to the far-field approach [*PRF*_FF,m_ calculated from Equation (11)].

In the upper part of Figure 9, the ratios of theoretical maximum power density obtained using the far-field and the near-field approaches are shown. The lower part shows the ratios between PRFs obtained using the near-field and far-field approaches. On one hand, the PRFs at closer distances are higher than those derived from the far-field approach, in line with Figure 7. On the other hand, the power density levels computed using the far-field formula [Equation (3)] are significantly higher than the true power density levels computed using the near-field approach [Equation (1)]. Therefore, the far-field approach still provides a conservative estimate of the actual maximum exposure levels. Table 2 compares the general public front compliance distance by using the results shown in Figure 8. The compliance distance results are rounded up to the nearest decimeter.

**Table 2:** Front compliance distances obtained using different approaches and for general public exposure limits (results are rounded up to the nearest decimeter).

	**ICNIRP 1998**	**ICNIRP 2020**
Far-field approach (PRF = 1)	2.0 m	1.1 m
Far-field approach (PRF = 0.32)	1.1 m	0.6 m
Far-field approach (PRF = 0.15 or 0.12)	0.8 m	0.4 m
Near-field approach, actual maximum exposure	0.8 m	0.4 m

**Figure 10:**
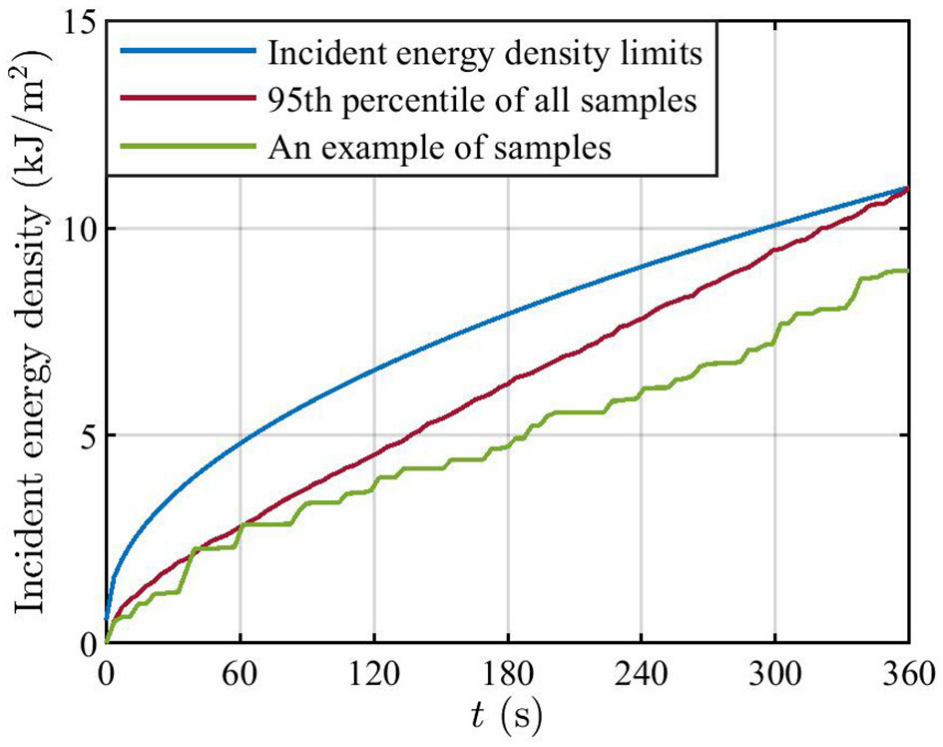
The blue curve is the incident energy density limits at 28 GHz. The red curve is the 95th percentile of normalized incident energy density at the position where the peak time-averaged power density occurs at *x* = 400 mm. The green curve is one example of samples at the same position. The curves for the 95th percentile and the sample are normalized such that the 95th percentile of incident energy density at 360 s is equal to the limit value at 360 s.

Figure 10 presents the Monte Carlo analysis results concerning the incident energy density limits. The blue curve is the incident energy density limits at 28 GHz. The position giving the actual maximum power density at *x* = 0.4 m is chosen to illustrate the incident energy density computed through the Monte Carlo analysis. The assumptions at the basis of such analysis are provided in the Supplementary Material. To illustrate the potential implications of the brief exposure limits on actual maximum exposure, the incident energy density results from the Monte Carlo analysis are also normalized in the figure, such that the 95th percentile of incident energy density at *t* = 360 s is equal to the limit value at *t* = 360 s (the red curve). The red curve is below the respective limit for intervals shorter than 360 s. This indicates that, according to the implemented model, the derived PRF based on the time-averaged power density ensures compliance also with the incident energy density limits on brief exposure. In addition, an example of Monte Carlo samples for normalized incident energy density is shown (the green curve). Similar figures can be drawn for other positions in space.

## Discussion

The PRF values are derived for the specific array antenna configuration used in this study. Usually, the PRF is reversely proportional to the number of antenna elements because antenna arrays with more elements have narrower beamwidth and a greater number of available beams in a codebook. This implies that, if larger antenna arrays are used in a mmW RBS, the PRFs derived from the array antenna used in this study may be applied to larger arrays with extra margin in actual maximum exposure. For RBSs enabling massive MIMO with 64 elements and operating below 6 GHz, a PRF of 0.32 is commonly used to determine the actual maximum exposure (8, 10). As indicated by Figure 8, applying a PRF of 0.32 for mmW RBSs will still lead to conservative results for the compliance boundary calculated from far-field antenna patterns.

The results in Figure 8 and Table 2 are obtained assuming *N* = 30 and *N* = 100 under the full-buffer condition. It should be emphasized again that these are very conservative assumptions when compared to real operating network measurements (15–17).

The EMF exposure limits for occupational exposure are five times higher than those for general public, resulting in smaller compliance boundaries. As indicated in Figure 8, at closer distances, the actual maximum exposure computed using the far-field approach is still larger than that computed using the near-field approach, suggesting that the mentioned far-field approach can also be applied to occupational exposure assessments.

The PRF derived in this study based on the near-field approach are applicable to mmW RBS transmitting at power levels <1 W, for which the whole-body exclusion criteria apply. As Figure 8 shows that the far-field approach is sufficiently accurate for distances larger than 0.5 m, for mmW RBSs operating above 1 W, the PRF that was derived based on the far-field approach may still be applicable.

Although the TDD downlink duty cycle was considered in the peak total EIRP calculation in this study, the derived PRF values do not include the effects of the TDD downlink duty cycle. If EIRP or power density values are provided for the maximum configured power level, the TDD downlink duty cycle should be considered in the calculation.

In this work, the actual maximum exposure is assessed using the ICNIRP incident power density limits. In the ICNIRP 2020 guidelines, a new exposure metric, absorbed power density, is introduced for local exposure above 6 GHz. The absorbed power density is directly related to the incident power density by the reflection coefficient of the exposed object. Therefore, the same PRFs as derived in this study are deemed to be applicable.

Below 6 GHz, measurement campaigns have confirmed that the actual maximum approach established using statistical models is conservative. Experimental studies conducted within 5G mmW live networks are also needed to validate the PRF levels derived in this study.

## Conclusions

In this article, a case study has been presented assessing the actual maximum exposure and PRFs for a mmW RBS characterized by lower EIRP levels compared to massive MIMO RBSs operating below 6 GHz. The results suggest that applying the PRF derived from a far-field approach to the far-field formula provides conservative power density levels even when the evaluation distance is close to the RBS antenna. The used workflow can be applied to other mmW RBSs and for other antenna configurations.

## Data Availability Statement

The original contributions presented in the study are included in the article/Supplementary Materials, further inquiries can be directed to the corresponding author.

## Author Contributions

BX conceived the method, wrote the code script for the Monte Carlo analysis, analyzed the results, and wrote the manuscript. DA and BX created the simulation model and script. BX, DC, and CT conceived the project. All authors reviewed the work.

## Conflict of Interest

BX, DA, DC, and CT are employed by the company Ericsson AB.

## Publisher's Note

All claims expressed in this article are solely those of the authors and do not necessarily represent those of their affiliated organizations, or those of the publisher, the editors and the reviewers. Any product that may be evaluated in this article, or claim that may be made by its manufacturer, is not guaranteed or endorsed by the publisher.

## Footnotes

^1^For RBSs with transmitted power less than 1 W, the whole-body–specific absorption rate (SAR) is below the whole-body SAR limit for general public (0.08 W/kg) by conservatively assuming that all power is absorbed by the body with a mass of 12.5 kg for a 4-year-old child, as described in Ref (4).
