# Supplementary material for: A Monte Carlo Analysis of Actual Maximum Exposure From a 5G Millimeter-Wave Base Station Antenna for EMF Compliance Assessments
Source: Front Public Health. 2022 Jan 7;9:777759. doi: 10.3389/fpubh.2021.777759 (PMC8777231; doi:10.3389/fpubh.2021.777759)
Supplement: Supplementary file 1 [file Image_1.pdf]

Supplementary Material

1 Comparison Between the Projected Poynting Vector and the Magnitude of the Poynting Vector

The actual maximum power density presented in Figures 8A and 8B is also computed using the magnitude of the Poynting vector [see Equation (2)] with the same random seeds. The relative differences between the magnitude and the x-component (the component projected to the direction normal to the evaluation planes) of Poynting vector are shown in Supplementary Figure 1. As can be seen from the figure, the relative differences between using Equation (3) and Equation (1) are less than 5% for all investigated distances, less than 3% for $d \geq 200$ mm, and less than 2% for $d \geq 500$ mm.

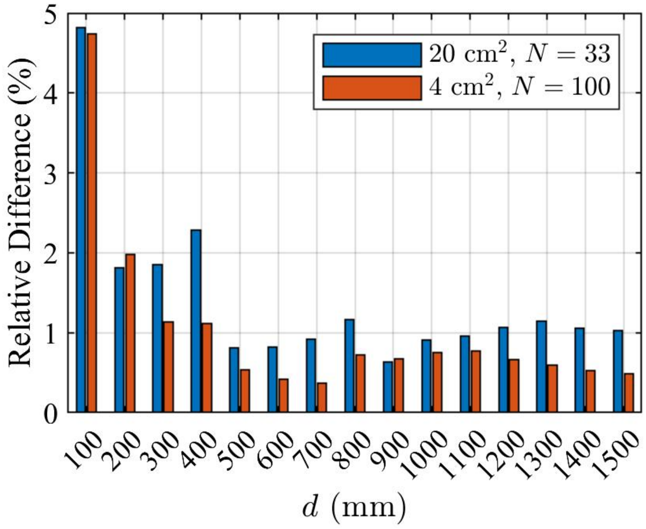

Supplementary Figure 1. Relative differences of actual maximum spatially averaged power density $S_{\mathrm{av}}(d)$ between using the magnitude of the Poynting vector [see Equation (3)] and the x-component of the Poynting vector [see Equation (1)] at different d. The blue bars correspond to the differences of actual maximum 20 cm^2 averaged power density, and the red bars correspond to the differences of actual maximum 4 cm^2 averaged power density.

2 Monte Carlo Analysis Considering Brief Exposure

For the ICNIRP 2020 guidelines, the Monte Carlo method described in the article is used for the computation of time-averaged power density over 6 minutes. Equation (7) generates the probability mass function, and Equation (8) provides the randomly generated N_l, $l = 1,2, \dots c$. Note that $\{N_l\}$ is the number of served UEs over 6 minutes, and how each UE is scheduled over 6 minutes is not assumed. A similar method is applied to the analysis of brief exposure based on the following additional assumptions:

1) The total time period related to brief exposure, i.e., 6 minutes (360 seconds), is equally divided by N.

2) In each time interval, i.e., $360/N$ seconds, only one UE is scheduled by one beam with full buffer. If $N = 100$ and the RBS downlink throughput is assumed to be 4.3 Gbps [1], it implies that one UE receives 15.5 Gb in 3.6 seconds, which is an extreme data usage scenario. For comparison, a one-hour 4K movie streaming would generate 7 GB [2].
3) The probability of the lth beam to be selected in one time interval still follows the multinominal distribution using the same probability distribution [see Equation (7)]. However, as only one beam is to be selected, the multinominal distribution becomes $\Pr\{X = l\} = \mathrm{Multinom}(1, \{p(l)\}), l = 1,2, \ldots, c$, where $X = l$ is the case that the lth beam is selected. This is a special case of multinomial distribution and is also called the categorical distribution.
4) In different time intervals, scheduling is independent. Over 6 minutes, a random sequence of the selected beams is generated.
5) For Monte Carlo analysis, 1000 samples are generated with each sample consisting of an aforementioned random sequence.

Obviously, for 6 minutes, the number of UEs served by the lth beam is the same as N_l. Therefore, the incident energy density results and their statistical properties obtained at the end of 6 minutes with the above assumptions are aligned with the time-averaged power density results presented in the paper. The illustration of the mentioned beam selection process is shown in Supplementary Figure 2A, and the corresponding incident energy density calculation for one Monte Carlo sample can be found in Supplementary Figure 2B.

For every time interval, each sample of the Monte Carlo analysis is characterized by a specific energy density level. Thus, the CDF curves and the 95th percentile of incident energy density are determined for each time interval. The 95th percentile results and an example of samples are given in Figure 10. Note that in Figure 10, the incident energy density values are normalized such that the 95th percentile is equal to the incident energy density limit value at 6 minutes, as described in the paper.

References

[1] How to control how much data Netflix uses (2021). Available online at: https://help.netflix.com/en/node/87

[2] Ericsson researchers top 4.3Gbps downlink on 5G millimeter Wave (2020). Available online at: https://www.ericsson.com/en/news/2020/2/ericsson-achieves-record-5g-mmwave-speed

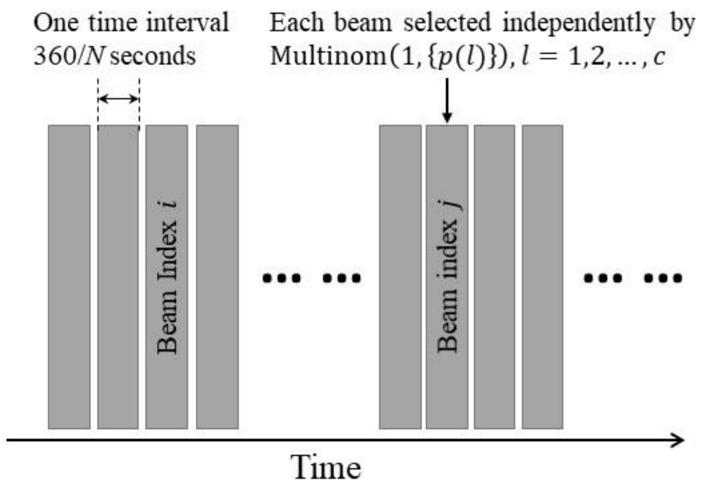

(A)

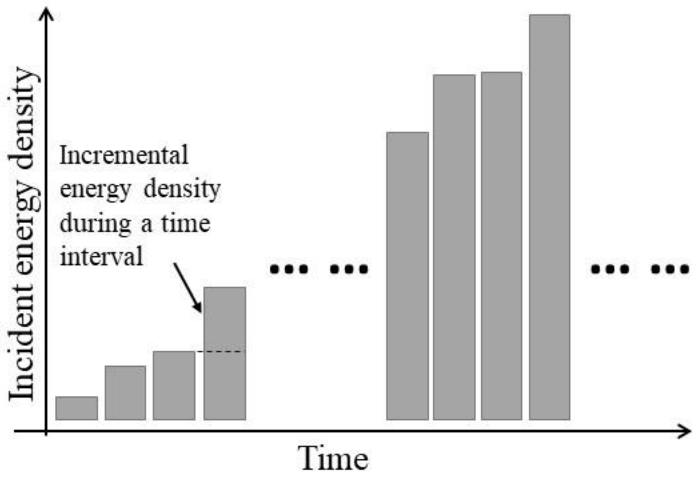

(B)

Supplementary Figure 2. (A) Illustration of randomly generated beams for every $360/N$ seconds. In each time interval, the RBS uses all downlink resources in one beam, which is determined by the categorical distribution. (B) Illustration of incident energy density calculation at a particular position in space. At each time interval, the incremental incident energy density generated by the scheduled beam is added to $U(t)$.
